# Dataset on the degradation of losartan by TiO_2_-photocatalysis and UVC/persulfate processes

**DOI:** 10.1016/j.dib.2020.105692

**Published:** 2020-05-14

**Authors:** John F. Guateque-Londoño, Efraím A. Serna-Galvis, Javier Silva-Agredo, Yenny Ávila-Torres, Ricardo A. Torres-Palma

**Affiliations:** a Grupo de Investigación en Remediación Ambiental y Biocatálisis (GIRAB), Instituto de Química, Facultad de Ciencias Exactas y Naturales, Universidad de Antioquia UdeA, Calle 70 No. 52-21, Medellín, Colombia; b Maestría en Ciencias Químicas, Facultad de Tecnología, Universidad Tecnológica de Pereira, Pereira, Colombia; c Grupo de Investigación QUIBIO, Facultad de Ciencias Básicas, Universidad Santiago de Cali, Santiago de Cali, Pampalinda, Colombia

**Keywords:** Antihypertensive elimination, Photochemical advanced oxidation processes, Pollutants degradation, Urine treatment, Water decontamination

## Abstract

Losartan is a highly consumed antihypertensive worldwide and commonly found in effluents of municipal wastewater treatment plants. In the environment, losartan can promote harmful effects on organisms. Thus, an option to face this pollutant is the treatment by photochemical advanced oxidation processes. This dataset has two main components: 1) theoretical calculations on reactivity indexes for losartan, and 2) degradation of the pollutant throughout TiO_2_-photocatalysis and UVC/persulfate (UVC/PS). The first part of the work presents the data about HOMO and LUMO energies, optimized geometry, dipolar moment, HOMO/LUMO energy gap and total density distribution, in addition to ionization energy, electron affinity, chemical potential, hardness, softness and electrophilicity for losartan. Meanwhile, the second one depicts information on the routes involved in the degradation of the pharmaceutical by the oxidation processes, mineralization, toxicity evolution and losartan removal from a complex matrix (synthetic fresh urine). The data reported herein may be utilized for further researches related to elimination of pharmaceuticals in primary pollution sources such as urine. Moreover, this work also provides experimental and theoretical data useful for the understanding of the response of losartan to oxidative and photochemical processes.

Specifications table

**Table:** 

Subject	Environmental chemistry
Specific subject area	Advanced oxidation process
Type of data	Table Figure
How data were acquired	Data were acquired by using HPLC-DAD and Gaussian 09 (software of quantum chemistry), Method: ground state, DFT, B3LYP, Basis: 6-311g ++ (2d, 2p).
Data format	Raw Analyzed
Parameters for data collection	The experiments were carried out at fixed operational conditions to establish the capability of TiO_2_-photocatalysis and UVC/Persulfate to degrade a highly consumed antihypertensive.
Description of data collection	The degradation at lab-scale of the antihypertensive losartan (LOS) by two photochemical process was performed. Initially, computational calculations on LOS were carried out. Then, the treatment in distilled water was done and the routes of process action were determined by using scavengers. Afterwards, data about mineralization and toxicity evolution were obtained. Finally, the information on matrix effect by LOS degradation in synthetic fresh urine was attained.
Data source location	Universidad de Antioquia UdeA, Medellín, Colombia; Universidad Santiago de Cali, Cali, Colombia; Universidad Tecnológica de Pereira, Pereira, Colombia
Data accessibility	Mendeley data repository through the following link: https://data.mendeley.com/datasets/7pbnd4vvm5/draft?a=a3dc88ff-086e-4baf-93b6-ab49d900e8cd

## Value of the data

• Data are useful to analyze similarities and differences between TiO_2_-photocatalysis and UVC/Persulfate for degrading pharmaceuticals such as losartan antihypertensive.
• Data can benefit people researching on elimination of antihypertensives by photochemical advanced oxidation processes in aqueous matrices.
• Data can be utilized for further insights about degradation of pharmaceuticals in a complex matrix, such as hospital effluents.
• Data are valuable for future works on oxidation processes, photochemistry and organic reactions of losartan.

## 1 Data Description

Dataset presented in this work have two main parts, the first component deals with computational calculations on losartan and the second one contains information about the degradation of the pharmaceutical by two advanced oxidation processes (i.e., TiO_2_-photocatalysis and UVC/persulfate). These photochemical processes are widely used for degrading organic pollutants in aqueous matrices [1], [2], [3], [4].

It should be mentioned that for double bonds in alkenes and aromatic rings (as contained in losartan structure), frontier orbitals (i.e., highest occupied molecular orbital-HOMO and in the lowest unoccupied molecular orbital-LUMO) can be useful to predict radical attack positions [5]. Then, energies of HOMO and LUMO, in addition to optimized geometry, dipolar moment, HOMO/LUMO energy gap and total density distribution, were theoretically stated, this information is presented in Table 1, Table 2. Meanwhile, Table 3 contains other reactivity indexes (such as ionization energy, electron affinity, chemical potential, hardness, softness and electrophilicity) for losartan.

**Table 1:** Computational calculations for losartan.

Parameter	**Results**
**Chemical structure of losartan**	
**Total energy**	
**Dipolar moment**	
**Highest occupied molecular orbital (HOMO)**	
**Lowest unoccupied molecular orbital (LUMO)**	
**Gap energy (E_GAP_)**	2.02 eV
**Total density distribution**

**Table 2:** Total density distribution for losartan.

**Moiety**	**Atom**	**Densities**	**Atoms numeration**
**Imidazole**	1 C	-0.013722	
	2 C	0.123255	
	3 C	-2.42648	
	4 N	0.053696	
	5 H	0.179991	
	6 N	0.310516	
**Chlorine**	7 Cl	-0.046173	
**Alcohol**	8 C	-0.72382	
	9 H	0.101355	
	10 H	0.143965	
	11 O	-0.458425	
	12 H	0.3764	
**Biphenyl**	13 C	0.294884	
	14 H	0.099872	
	15 H	0.102713	
	16 C	0.548818	
	17 C	-0.382691	
	18 C	-0.470243	
	19 C	-0.512244	
	20 H	0.136655	
	21 C	-0.637257	
	22 H	0.150577	
	23 C	0.667093	
	24 H	0.168494	
	25 H	0.175748	
	26 C	0.391657	
	27 C	0.446463	
	28 C	-0.177684	
	29 C	-0.194079	
	30 C	-0.374148	
	31 H	0.169917	
	32 C	-0.404455	
	33 H	0.169726	
	34 H	0.160523	
	35 H	0.154444	
**Tetrazole**	36 C	-0.005707	
	37 N	-0.290324	
	38 N	-0.354756	
	39 N	0.004876	
	40 N	0.12792	
	41 H	0.302545	
**Butyl**	42 C	1.416066	
	43 C	-0.575332	
	44 H	-0.001771	
	45 H	0.034663	
	46 C	-0.046357	
	47 H	0.078481	
	48 H	0.05442	
	49 C	-0.479371	
	50 H	0.076031	
	51 H	0.069222	
	52 H	0.103527	
	53 H	0.090485	
	54 H	0.090042

**Table 3:** Reactivity indexes for losartan.

Ionization energy (eV)	Electron affinity (eV)	Chemical potential (eV)	Global Hardness (eV)	Local softness (eV)	Global index of electrophilicity (eV)
2.2005	2.1434	2.1719	0.0571	17.513	42.062

**Fig. 1:**
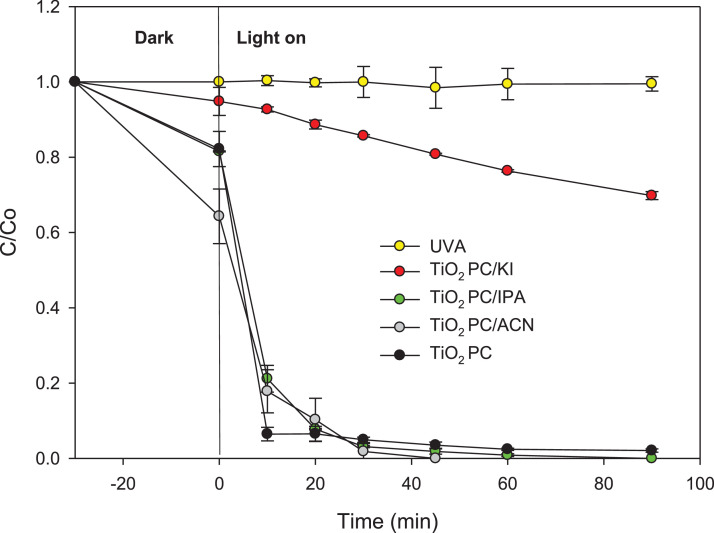
Degradation of losartan using TiO_2_-photocatalysis (TiO_2_ PC). [Losartan]= 43.38 µmol L^-1^, initial pH: 6.1, [TiO_2_] = 0.5 g L^-1^, [KI]= [IPA]= 4.33 mmol L^-1^ and UVA light power = 75 W.

**Fig. 2:**
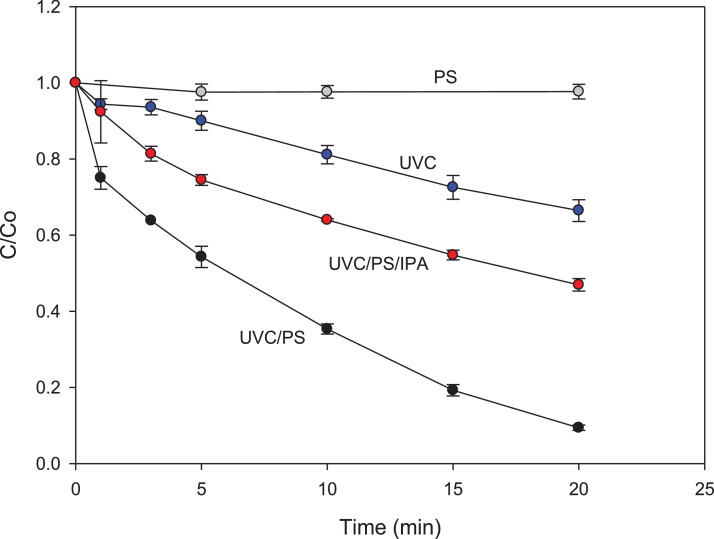
Degradation of losartan by the UVC/PS process. [Losartan]= 43.38 µmol L^-1^, initial pH: 6.1, [PS] = 500 µmol L^-1^, [IPA]= 4.33 mmol L^-1^ and UVC light power = 60 W.

Regarding losartan degradation by the AOPs, in Fig. 1 is shown the antihypertensive evolution during the treatment in distilled water using TiO_2_-photocatalysis (TiO_2_ PC). Fig. 1 also presents data on removal by photolysis (UVA), the pollutant degradation in presence of potassium iodide and isopropanol scavengers (TiO_2_ PC/KI and TiO_2_ PC/IPA, respectively) and replacing water media by acetonitrile solvent (TiO_2_ PC/ACN) to provide information about the routes involved in the process [1,2]. In turn, Fig. 2 presents the degradation of losartan by the UVC/PS system, control experiments (action of persulfate-PS or the light-UVC), plus the dataset for experiments when isopropanol (UVC/PS/IPA, which is a scavenger of hydroxyl and sulfate radicals [6]) is added.

**Fig. 3:**
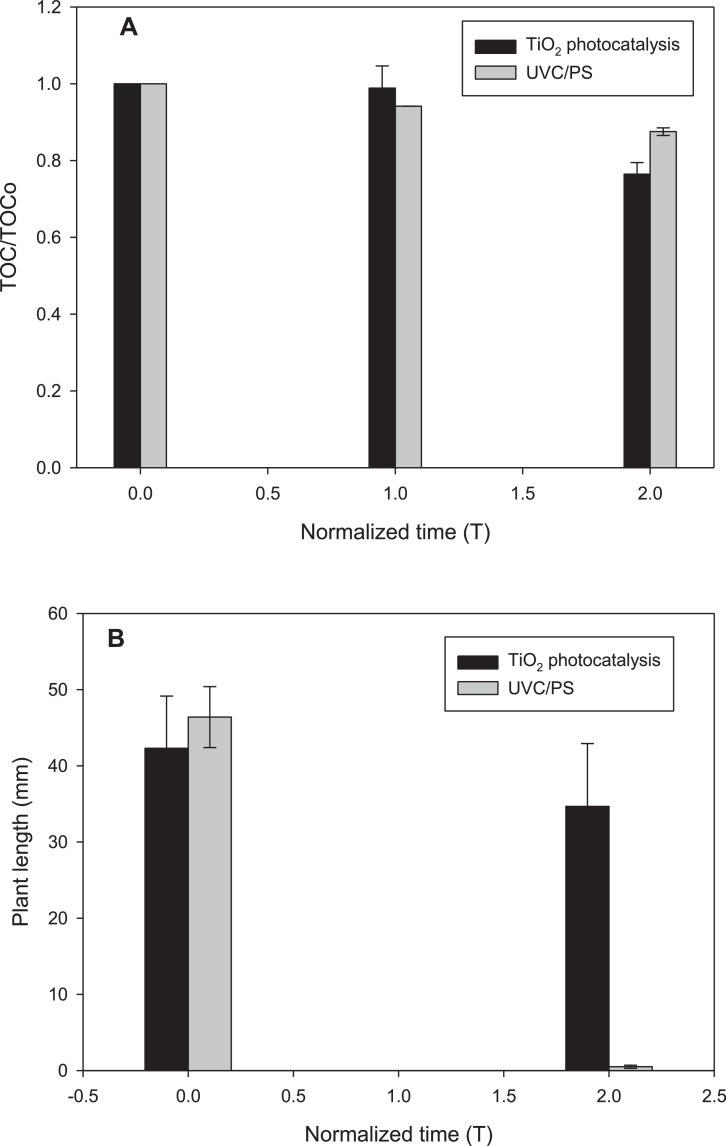
Extension of advanced oxidation treatments. **A.** Mineralization of losartan during application of the different processes. **B.** Toxicity against radish seeds (*Raphanus sativus*) of treated solution of losartan. Experimental conditions as described in Figs. 1 and 2.

**Fig. 4:**
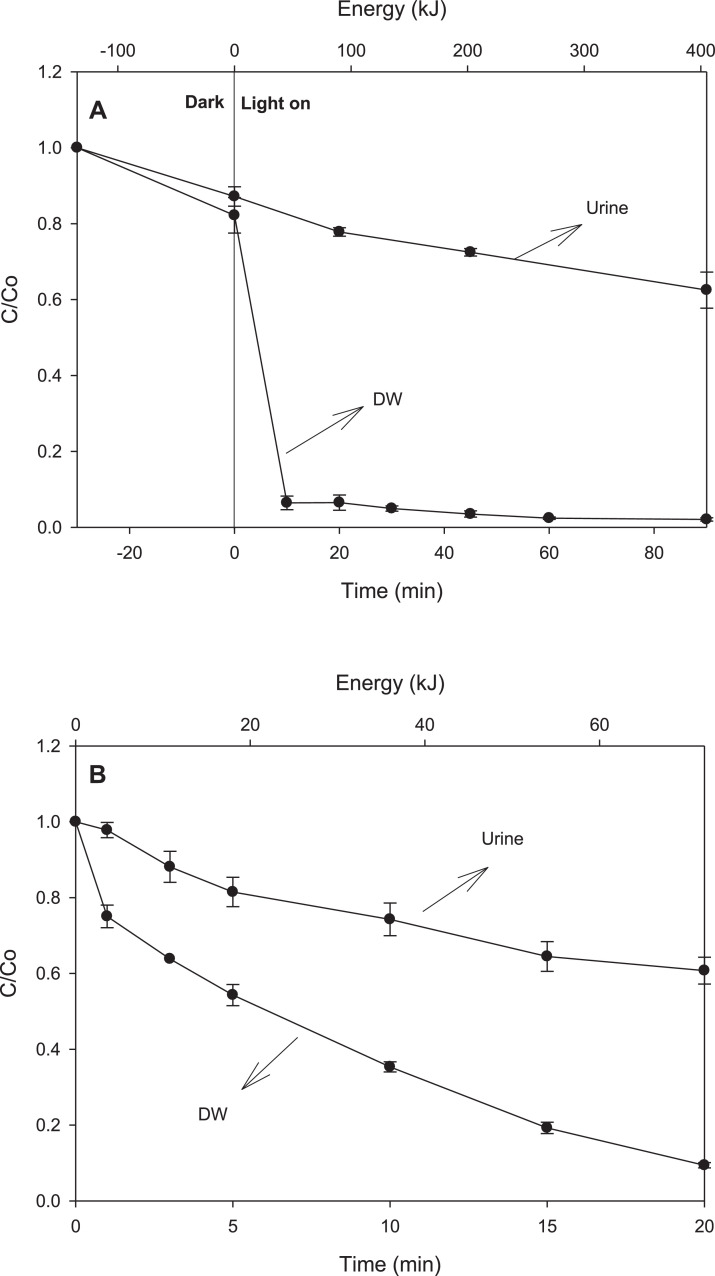
Comparison of losartan degradation in distilled water (DW) and simulated fresh urine (Urine). **A.** TiO_2_ photocatalysis. **B.** UVC/PS process. Experimental conditions as described in Figs. 1 and 2.

In Fig. 3 is presented the evolution of total organic carbon (TOC) and phytotoxicity under the two processes, for comparative purposes, the TOC removal (Fig. 3A) and toxicity (Fig. 3B) were measured at two normalized times: 1 (when losartan is 100% degraded) and 2 (the double of time required to 100% remove the antihypertensive). Fig. 4. compares the treatment of losartan in distilled water and synthetic fresh urine by TiO_2_-photocatalysis (Fig. 4A) and UVC/PS (Fig. 4B) processes. Table 4 depicts the synthetic fresh urine composition and Table 5 summarizes the literature search on the interaction/reaction among hydroxyl or sulfate radicals with the urine components, in addition to the pseudo-first order rate constants for losartan degradation by TiO_2_-photocatalysis and UVC/PS.

**Table 4:** Composition of synthetic fresh urine ([7]) used for the experiments.

**Compound**	**Concentration (mol L^-1^)**
Urea	0.2664
CH_3_COONa	0.1250
Na_2_SO_4_	0.01619
NH_4_Cl	0.03365
NaH_2_PO_4_	0.02417
KCl	0.05634
MgCl_2_	0.003886
CaCl_2_	0.004595
NaOH	0.00300
pH = 6.1

**Table 5:** Rate constants of the reactions between the radical species and the components of fresh urine.

Reaction	Second order rate constant (k^2nd^, L mol^-1^ s^-1^)	References
$HO^{•}+Cl^{-}\to \;ClOH^{•-}$	4.3x10^9^	[8]
$HO^{•}+\;H_{2}PO_{4}^{-}\to HO^{-}+H_{2}PO_{4}^{•}$	∼ 2x10^4^	[9]
$HO^{•}+\;CH_{3}COO^{-}\;\to H_{2}O+CH_{2}COO^{•-}$	7.0 x10^7^	[10]
$HO^{•}+OH^{-}\to \;O^{•}+H_{2}O$	1.3x10^10^	[11]
$HO^{•}+H_{2}NCONH_{2}\to products\;$	7.9x10^5^	[9]
$HO^{•}+\;SO_{4}^{2-}\;\to \;SO_{4}^{•-}+\;HO^{-}$	6.5x10^2^	[3]
$SO_{4}^{•-}+Cl^{-}\to SO_{4}^{2-}+Cl^{•}$	3.1×10^8^	[4]
$SO_{4}^{•-}+OH^{-}\to SO_{4}^{2-}+HO^{•}$	6.5x10^7^	[12]
$SO_{4}^{•-}+NH_{4}^{+}\;/\;NH_{3}\;\to \;products\;$	3.5×10^5^	[13]
$SO_{4}^{•-}+CH_{3}CO_{2}^{-}\to SO_{4}^{2-}{+}^{•}CH_{3}+CO_{2}\;({+}^{•}CH_{2}CO_{2}^{-}\;$)	5.8x10^6^	[14]
$SO_{4}^{•-}+H_{2}PO_{4}^{-}\to products$	< 7x10^4^	[13]
**Pseudo-first order rate constant (k, min^-1^) for degradation of losartan by the processes**		
TiO_2-_photocatalysis	0.004	In this work
UV/PS	0.029	In this work

## 2 Experimental Design, Materials, and Methods

### 2.1 Reagents

Acetonitrile, isopropanol, methanol, potassium iodide, potassium persulfate, sodium acetate, sodium chloride, sodium dihydrogen phosphate, sodium hydroxide, sodium sulfate, and urea were provided by Merck. Ammonium chloride, formic acid, calcium chloride and magnesium chloride were provided by PanReac. Titanium dioxide was provided by Evonik. Losartan was purchased from La Santé S.A. The solutions of losartan were prepared using distilled water. In all cases, the initial losartan concentration was 43.38 µmol L^-1^.

### 2.2 Reaction systems

A homemade aluminum reflective reactor containing UVC lamps (OSRAM HNS®, 60 W of light power) with main emission at 254 nm was used for the UVC/PS process. Losartan solutions (50 mL) were placed in beakers (100 mL of capacity) under constant stirring. The TiO_2_-photocatalysis process was carried out in the same reactor but equipped with UVA lamps (Philips BLB, 75 W of light power) having main emission peak at 365 nm. Losartan solutions (50 mL) were also placed in beakers under constant stirring. Additionally, the adsorption/desorption equilibrium on TiO_2_ catalyst was reached after 30 min in dark.

Aliquots of 0.5 mL were taken periodically from the rectors for kinetics analyses by UHPLC (no more than nine aliquots were considered to avoid modifications of the sample volume higher than 10%). For total organic carbon and toxicity measurements, independent experiments were performed and the whole sample was considered in each case per point of the analyses.

### 2.3 Analyses

Losartan evolution was determined by means a UHPLC Thermo Scientific Dionex UltiMate 3000 chromatograph equipped with an Acclaim™ 120 RP C18 column (5 µm, 4.6 x150 mm) and a DAD (operated at 230 and 254 nm). The mobile phase was methanol/acetonitrile/formic acid (10 mM and pH 3.0) at 10/44/46 %v/v at 0.6 mL min^-1^.

Mineralization was established using 10 mL of sample by measuring of total organic carbon (TOC), through a Shimadzu LCSH TOC analyzer (previously calibrated), according to Standard Methods 5310, by combustion with catalytic oxidation at 680 °C using high-purity oxygen gas at a flow rate of 190 mL/min. The apparatus had a non-dispersive infrared detector.

Toxicity against radish seeds (*Raphanus sativus)* was established by interaction of target solution with the indicator seeds. The solution to be tested (5 mL) was placed in a petri dish; then, ten (10) *Raphanus sativus* seeds were submerged into the solution. The seeds and solution were in contact during 72 h. Afterward, the length of germinated plants was measured, subsequently a mean value and standard deviation for each tested solution were calculated.

The computational calculations were done by using Gaussian 09 (quantum chemistry software); Method: ground state, DFT, B3LYP; Basis: 6-311g ++ (2d, 2p) [15]. The neutral molecule was considered using the dielectric constant for water.

## Appendix Supplementary materials

Image, application 1

Image, application 2
